# Relative Melatonergic Status and Systemic Inflammatory, Oxidative, and Biological Aging Burden in Adults: An Exploratory Cross-Sectional Biomarker Analysis

**DOI:** 10.3390/cancers18162597

**Published:** 2026-08-12

**Authors:** Alexandre Tavartkiladze, Levan Tavartkiladze, Russel J. Reiter, Michel Burnier, Engin Ulukaya, Revaz Turmanidze, Pati Revazishvili

**Affiliations:** 1 Department of Medical Oncology and Cellular Therapy, Faculty of Medicine, Tbilisi State Medical University, Tbilisi 0186, Georgia; 2 Institute for Personalized Medicine of Georgia (PMI), Tbilisi 0186, Georgia; levan001@gmail.com (L.T.); revaz555@hotmail.com (R.T.); pati_revazishvili@yahoo.com (P.R.); 3 Department of Cellular & Structural Biology, The University of Texas Health Science Center at San Antonio, San Antonio, TX 78229, USA; reiter@uthscsa.edu; 4 Service of Nephrology and Hypertension, Lausanne University Hospital (CHUV), University of Lausanne, 1011 Lausanne, Switzerland; michel.burnier@netplus.ch; 5 Faculty of Medicine, Istinye University, Istanbul 34396, Turkey; eulukaya@istinye.edu.tr

**Keywords:** melatonin, 6-sulfatoxymelatonin, aMT6s, inflammation, oxidative stress, antioxidant defense, biological aging, biomarker composite, circadian biology, cross-sectional analysis

## Abstract

Background/objectives: Melatonin has circadian, antioxidant, mitochondrial, and immunomodulatory functions, but human data linking relative melatonergic status to coordinated systemic biomarker burden remain limited. We examined this association and tested its internal robustness to alternative score construction, outlier handling, and missing-data assumptions. Methods: We performed an adult-only cross-sectional secondary analysis of a deidentified biomarker dataset. Of 322 source records, six participants younger than 18 years and two records without age were excluded, yielding 314 adults. Plasma melatonin and 24 h urinary aMT6s were converted to within-cohort rank percentiles and averaged to form a relative melatonergic-axis score. Equal-weight z-score composites represented inflammation, oxidative damage, antioxidant deficit, and biological aging/biological injury; their mean was the integrated systemic biomarker-burden score. Analyses included Kruskal–Wallis tests; Spearman correlations with bootstrap confidence intervals; standardized regression adjusted for age, sex, and BMI; robust covariance estimates; principal component analysis (PCA), winsorization, leave-one-domain/marker-out analyses, and missing-BMI sensitivity models. Results: The lower, intermediate, and higher relative melatonergic tertiles included 105, 104, and 105 adults, respectively. The melatonergic-axis score correlated inversely with integrated systemic burden (Spearman ρ = −0.944; 5000-resample bootstrap 95% CI, −0.953 to −0.931; *p* < 0.001). In age-, sex-, and BMI-adjusted complete-case models (*N* = 250), each 1 SD higher axis score was associated with a 0.95 SD lower integrated burden (β = −0.949; HC3 95% CI, −0.983 to −0.915; *p* < 0.001). Results were similar for separate plasma melatonin and aMT6s models, winsorized composites, a PCA-derived score (PC1 explained 75.5% of marker variance; ρ = −0.947), leave-one-domain/marker-out analyses, and missing-BMI sensitivity models. Conclusions: Lower relative melatonergic status was associated with a highly coordinated adverse systemic biomarker pattern. The exceptional magnitude of the association requires audit of primary assay provenance and independent external replication. Because cancer, microbiome, and liquid biopsy endpoints were not measured, oncology implications remain untested prospective hypotheses.

## Simple Summary

This exploratory cross-sectional secondary analysis evaluated relative melatonergic status, represented by plasma melatonin and 24 h urinary 6-sulfatoxymelatonin (aMT6s), tracked with a coordinated systemic biomarker pattern in adults. Lower within-cohort melatonergic scores were associated with higher inflammatory and oxidative damage burden, weaker antioxidant defense, and higher biological aging/biological injury burden. The association was internally stable across alternative score constructions, robust covariance estimates, outlier handling, leave-one-out analyses, and missing-BMI sensitivity analyses. However, the source dataset did not include standardized circadian sampling metadata, complete assay provenance, major clinical confounders, cancer outcomes, microbiome sequencing, or CTC/cfDNA/ctDNA measurements. Accordingly, the findings do not establish causality, cancer risk, diagnostic accuracy, or benefit from melatonin supplementation. They define a hypothesis-generating systemic phenotype that requires independent longitudinal validation.

## 1. Introduction

Cancer develops through interacting processes that include genomic instability, chronic inflammation, altered metabolism, immune dysregulation, and microenvironmental selection [1,2]. Reactive oxygen and nitrogen species can contribute to DNA, protein, and lipid damage while also functioning as signaling mediators; persistent redox imbalance and inflammatory activation are therefore biologically relevant to carcinogenesis, aging, and tissue injury [3,4,5,6,7,8,9,10,11]. These relationships do not imply that a nonspecific inflammatory or oxidative biomarker panel can diagnose cancer, but they motivate careful study of systemic biological states that may precede or accompany disease.

Melatonin is the canonical endocrine output of the central circadian system and also participates in mitochondrial homeostasis, radical scavenging, antioxidant enzyme regulation, and immune modulation [12,13,14]. Plasma melatonin is strongly dependent on clock time, light exposure, sleep timing, and collection conditions, whereas 24 h urinary 6-sulfatoxymelatonin (aMT6s) integrates melatonin metabolism across a longer interval. These measures are complementary but are not interchangeable, and neither establishes a clinical diagnosis of melatonin deficiency without validated sampling and reference procedures.

Microbiome and liquid biopsy variables occupy distinct translational layers. Microbial communities can influence epithelial barriers, metabolism, inflammation, immune tone, and treatment response, but microbiome–cancer associations are sensitive to sampling, sequencing methods, and confounding [15,16,17,18,19,20,21]. Circulating tumor cells (CTCs) and cell-free or circulating tumor DNA (cfDNA/ctDNA) are tumor-related markers used primarily for genotyping, treatment monitoring, recurrence risk, and molecular residual disease rather than as generic measures of systemic oxidative stress [22,23,24,25,26,27,28,29,30,31,32]. These layers should be separated conceptually unless they are measured together in a longitudinal protocol.

The present study therefore focuses only on variables directly available in an adult biomarker dataset: plasma melatonin, 24 h urinary aMT6s, and inflammatory, oxidative, antioxidant, and biological aging/biological injury markers. The primary objective was to test whether lower relative melatonergic status was associated with higher systemic biomarker burden. Secondary objectives were to assess the influence of equal-weight composite construction, missing BMI data, outliers, and alternative data reduction methods. Microbiome, cancer, CTC, cfDNA, and ctDNA endpoints are treated only as prospective research targets and not as outcomes of the present analysis.

## 2. Materials and Methods

### 2.1. Study Design, Source Dataset, and Analytic Population

This was an exploratory cross-sectional secondary analysis of a deidentified dataset derived from an existing Georgian family-based biomarker cohort. The source workbook contained 322 participant records and 124 family identifiers. Six participants younger than 18 years and two records without age were excluded; the adult analytic population therefore comprised 314 participants. No new recruitment, intervention, specimen collection, or participant contact occurred for this reanalysis.

**Figure 1:**
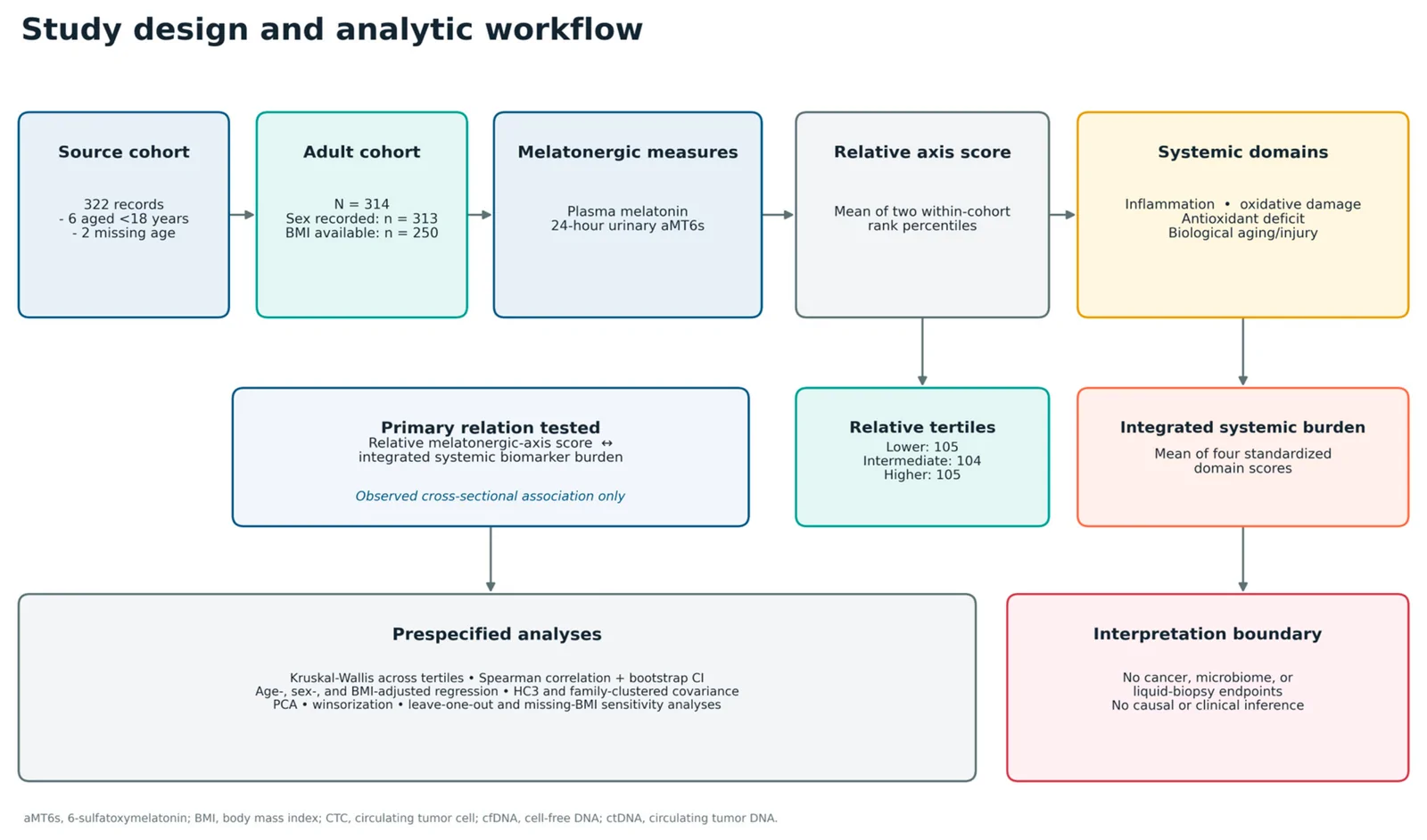
Study design, measured variables, analytic workflow, and interpretation boundary. Solid arrows denote derivation steps. The bidirectional symbol identifies the cross-sectional association tested; no causal direction is inferred. Variables listed in the interpretation-boundary box were not measured in the source dataset.

The analytic dataset contained age, sex, BMI, plasma melatonin, 24 h urinary aMT6s, inflammatory biomarkers, oxidative damage markers, antioxidant markers, and biological aging/biological injury indicators. It did not contain cancer diagnosis or outcome, tumor stage, recurrence, progression, treatment response, microbiome sequencing, CTC enumeration, cfDNA/ctDNA measurements, or prospective follow-up. Complete source recruitment criteria, comorbidity history, smoking, medication exposure, melatonin supplementation, shift-work status, sleep timing, and light-at-night exposure were not available in the analytic file. These omissions precluded clinical risk modeling and adjustment for those potential confounders (Figure 1).

### 2.2. Melatonin and aMT6s Variables and Measurement Metadata

Plasma melatonin (pg/mL) and 24 h urinary aMT6s (µg/24 h) were analyzed as recorded in the source dataset. The file did not include blood-collection clock time, ambient light conditions, sleep schedule, season, medication timing, assay manufacturer, calibration information, lower limit of detection, intra-assay or inter-assay coefficients of variation, batch or plate identifiers, sample storage duration, or freeze–thaw history. For urinary aMT6s, the file also lacked collection start/end times, completeness checks, urine creatinine, and collection-specific quality-control fields. Consequently, plasma melatonin was not interpreted as circadian phase or nocturnal amplitude, and the terms lower, intermediate, and higher refer only to relative positions within this cohort rather than to clinical deficiency or reference thresholds.

### 2.3. Relative Melatonergic-Axis Score and Tertiles

Plasma melatonin and urinary aMT6s were converted separately to within-cohort rank percentiles. The relative melatonergic-axis score was the arithmetic mean of the two percentiles. This rank-based approach placed the two assays on a common scale, reduced sensitivity to distributional skew and unit differences, and avoided selecting an unvalidated clinical cut-off. The score was divided at its empirical one-third and two-thirds quantiles into lower, intermediate, and higher relative melatonergic tertiles. Because the combined score could conceal measure-specific behavior, plasma melatonin and aMT6s were also analyzed separately in correlation and adjusted-regression sensitivity analyses.

### 2.4. Composite Biomarker Definitions

Each marker was z-standardized using the adult analytic mean and population standard deviation. The inflammation composite included GDF-15, suPAR, IL-1β, IL-6, TNF-α, hsCRP, and serum amyloid A. The oxidative damage composite included malondialdehyde (MDA), 8-hydroxy-2′-deoxyguanosine (8-OHdG), F2-isoprostanes, advanced oxidation protein products, protein carbonyls, 3-nitrotyrosine, total oxidant status, and oxidative stress index. The antioxidant-deficit composite inverted total antioxidant capacity/status, superoxide dismutase, glutathione peroxidase, catalase, reduced glutathione, and the GSH/GSSG ratio so that higher scores represented weaker antioxidant defense. The biological aging/biological injury composite included NT-proBNP, high-sensitivity troponin, neurofilament light, glial fibrillary acidic protein, GlycanAge, GrimAge, DunedinPACE, and DNA-methylation age acceleration. Domain scores were available-item means, and the integrated systemic biomarker-burden score was the equal-weight mean of the four domains.

Equal weighting was chosen a priori to avoid outcome-driven optimization in a dataset without a cancer endpoint and to keep the exploratory score transparent. The integrated score is not a validated diagnostic or prognostic scale. Its robustness was evaluated using a principal component score, 1st/99th percentile winsorization, leave-one-domain-out and leave-one-marker-out analyses, and separate melatonin/aMT6s models.

### 2.5. Statistical Analysis

Continuous variables are summarized as mean ± standard deviation unless otherwise stated, and categorical variables are summarized as counts and percentages. Differences across relative melatonergic tertiles were evaluated using Kruskal–Wallis tests. Associations between rank-based melatonergic measures and composite outcomes were evaluated with Spearman correlation. A percentile bootstrap with 5000 resamples estimated the 95% confidence interval for the primary correlation between the combined axis and integrated systemic burden.

Standardized linear regression models estimated the change in each standardized composite per 1 SD higher melatonergic-axis score, adjusted for age, sex, and BMI. The primary adjusted analysis was complete-case because BMI was missing in 64 adults. Conventional, HC3 heteroskedasticity-robust, and family-clustered covariance estimates were calculated. Variance inflation factors assessed collinearity among the axis, age, sex, and BMI. Missing-data sensitivity included comparison of the 250 complete cases with the 64 adults omitted from the BMI-adjusted model, an age-and-sex model among participants with recorded sex, and a model using mean-imputed BMI plus a missingness indicator.

Robustness analyses recomputed the integrated score after winsorizing each marker at the 1st and 99th percentiles, derived a principal component score from complete marker cases, repeated analyses after omitting each domain or marker, and modeled plasma melatonin and urinary aMT6s separately. All tests were two-sided. Because the study was exploratory and lacked a clinical endpoint, *p* values quantify statistical association rather than causality, diagnostic performance, or therapeutic effect. Analyses were performed in Python using NumPy, SciPy, and statsmodels.

## 3. Results

### 3.1. Adult Cohort and Relative Melatonergic Tertiles

The adult analytic population included 314 participants (median age, 50 years; range, 18–84 years). Sex was recorded as female in 192 participants and male in 121; one participant had missing sex coding. BMI was available in 250 participants. The lower, intermediate, and higher relative melatonergic tertiles contained 105, 104, and 105 participants, respectively. Mean plasma melatonin increased from 4.18 ± 1.99 pg/mL in the lower tertile to 22.46 ± 5.20 pg/mL in the higher tertile, while mean urinary aMT6s increased from 6.01 ± 2.29 to 32.97 ± 9.17 µg/24 h (Table 1).

### 3.2. Selected Inflammatory, Oxidative, Antioxidant, and Aging/Injury Gradients

Lower relative melatonergic status was accompanied by an adverse gradient across multiple measured biomarkers. Compared with the higher tertile, the lower tertile had higher hsCRP (5.05 ± 1.42 vs. 0.97 ± 0.56 mg/L), IL-6 (4.93 ± 1.61 vs. 1.18 ± 0.55 pg/mL), MDA (5.00 ± 0.56 vs. 2.54 ± 0.63 µmol/L), 8-OHdG (10.25 ± 1.47 vs. 4.49 ± 1.56 ng/mL), and oxidative stress index (29.34 ± 6.58 vs. 7.62 ± 3.75). The GSH/GSSG ratio increased from 11.32 ± 2.48 in the lower tertile to 60.74 ± 33.06 in the higher tertile. NT-proBNP and DunedinPACE also declined across the relative melatonergic gradient (Table 1).

**Figure 2:**
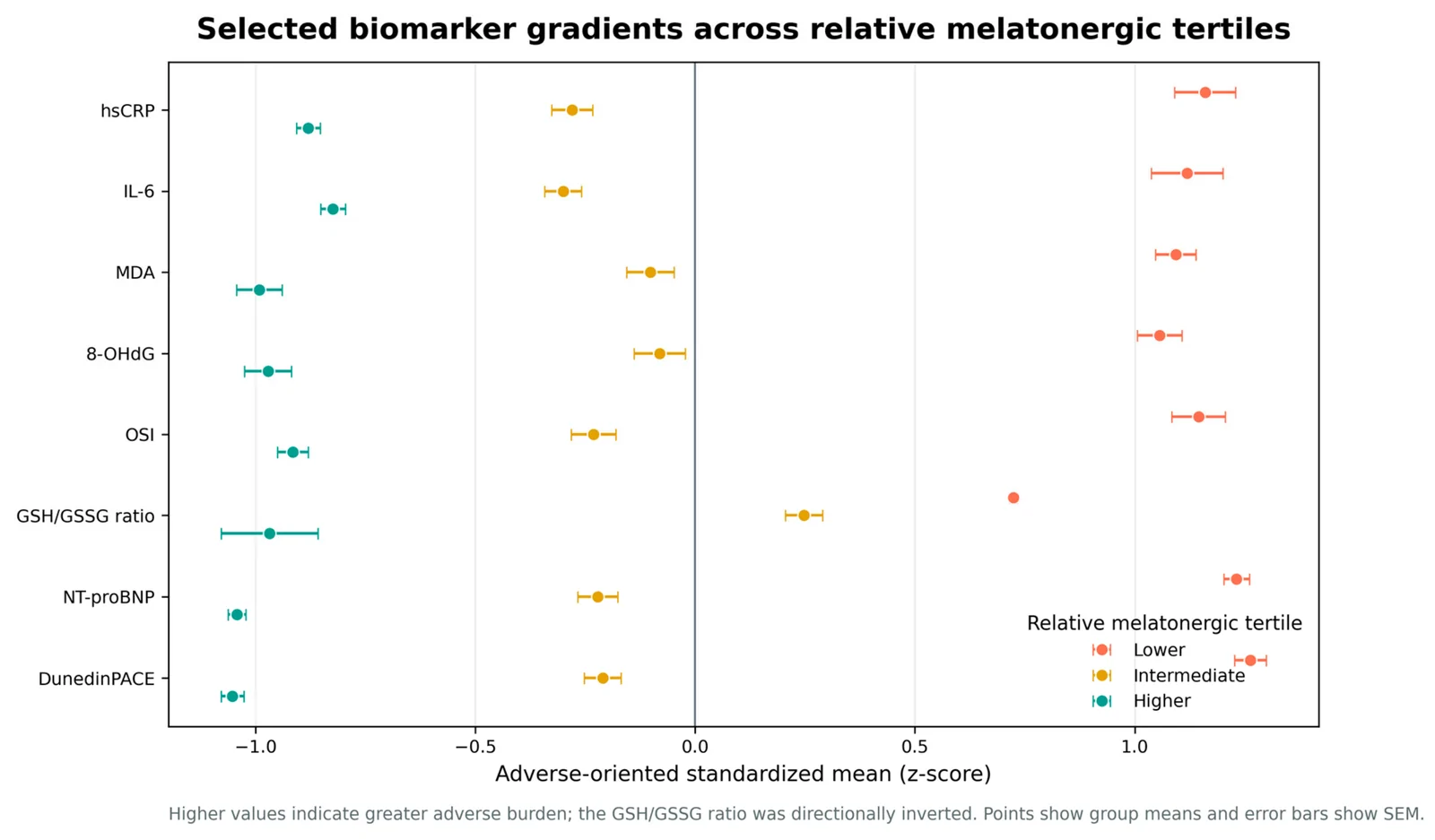
Selected biomarker gradients across relative melatonergic tertiles. Marker means were standardized against the overall adult mean and SD; higher values indicate greater adverse burden, and the GSH/GSSG ratio was directionally inverted. Points show group means, and error bars show SEM.

For visual comparison across unlike units, selected marker means were transformed to adverse-oriented z-scores using the overall adult mean and standard deviation; the GSH/GSSG ratio was directionally inverted. The resulting pattern was monotonic across lower, intermediate, and higher relative melatonergic tertiles (Figure 2). All selected continuous biomarkers differed across tertiles at *p* < 0.001.

### 3.3. Correlation and Adjusted Regression Analyses

**Figure 3:**
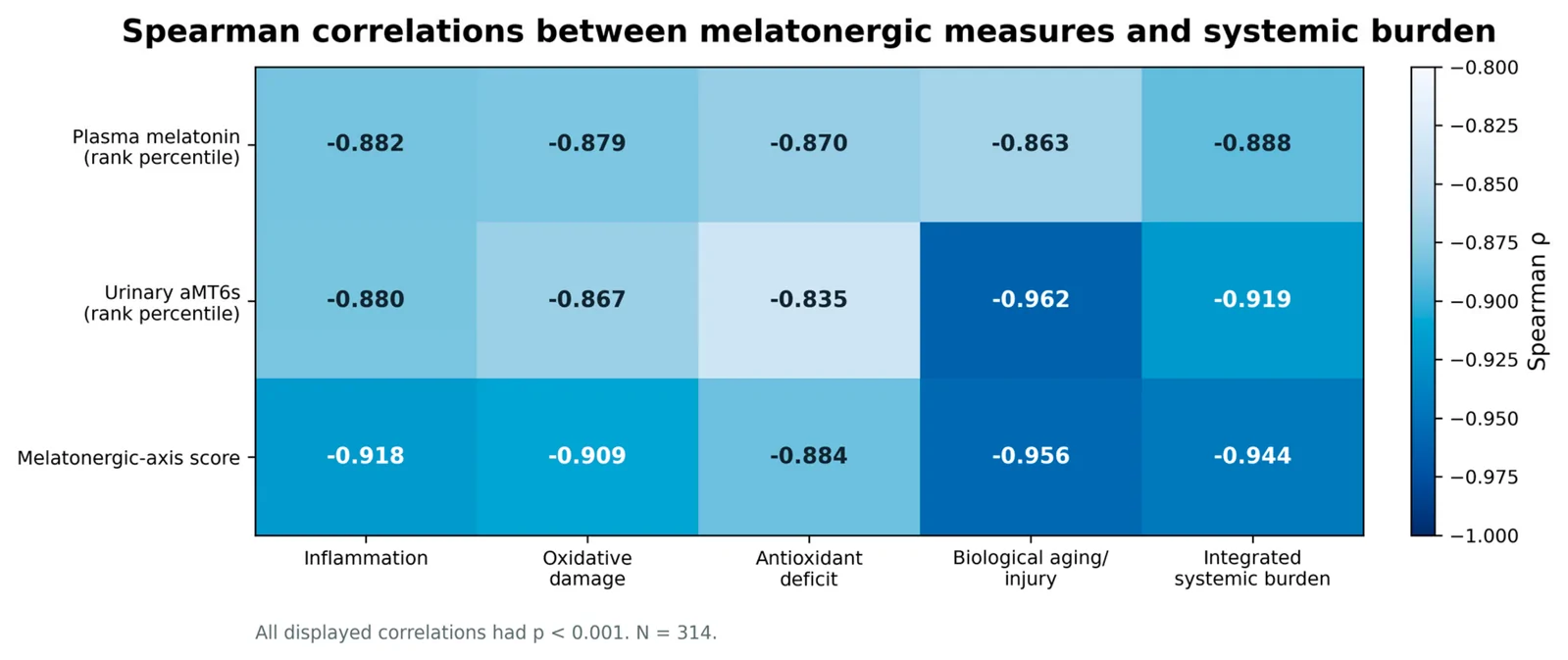
Spearman correlations between melatonergic measures and systemic composite burden. Plasma melatonin and aMT6s were analyzed as rank percentiles to retain scale comparability. All displayed correlations had *p* < 0.001; N = 314.

The melatonergic-axis score correlated strongly and inversely with integrated systemic burden (Spearman ρ = −0.944; bootstrap 95% CI, −0.953 to −0.931; *p* < 0.001). Corresponding correlations were −0.918 for inflammation, −0.909 for oxidative damage, −0.884 for antioxidant deficit, and −0.956 for biological aging/injury. Plasma melatonin and urinary aMT6s analyzed separately also correlated inversely with integrated burden (ρ = −0.888 and −0.919, respectively; Figure 3).

**Figure 4:**
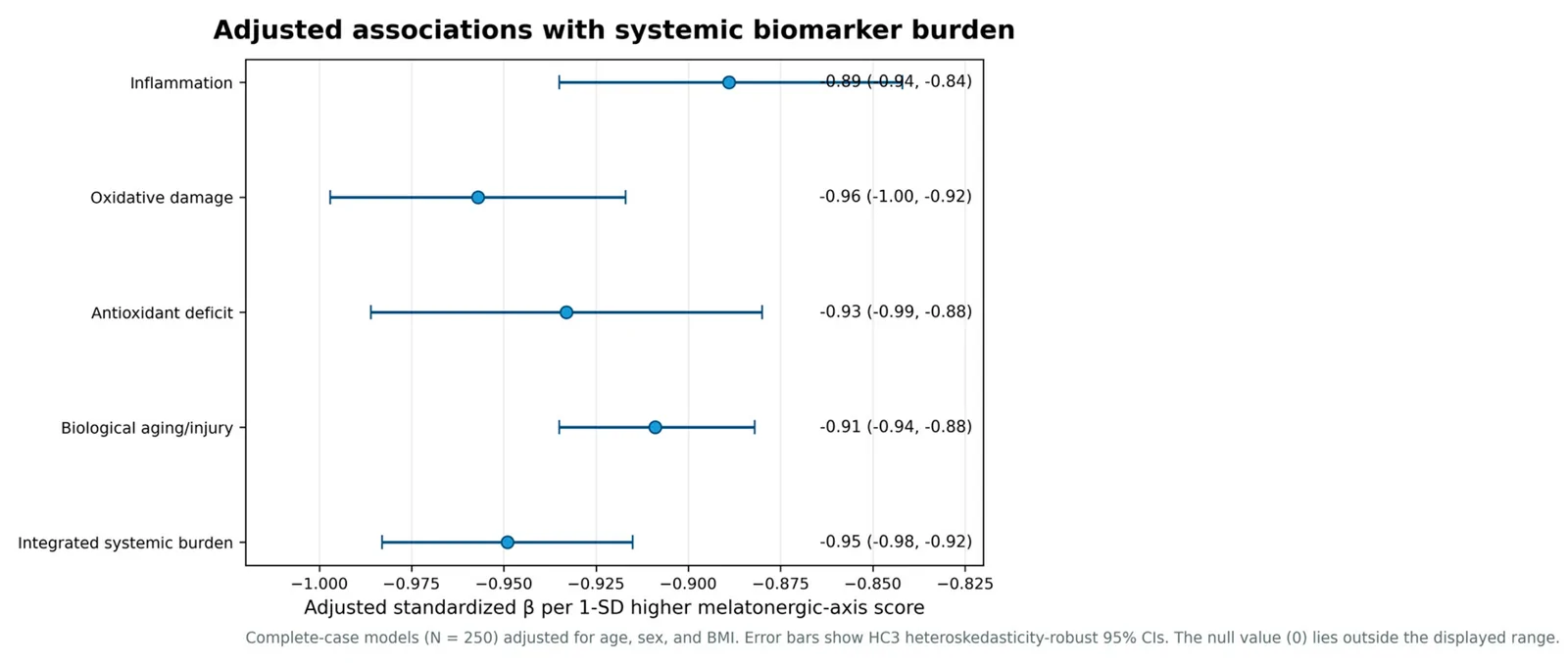
Adjusted associations with systemic biomarker burden. Points show standardized regression coefficients, and error bars show HC3 heteroskedasticity-robust 95% confidence intervals. Complete-case models (N = 250) were adjusted for age, sex, and BMI. The null value lies outside the plotted range.

In complete-case models adjusted for age, sex, and BMI (N = 250), each 1 SD higher melatonergic-axis score was associated with a 0.95 SD lower integrated systemic burden (β = −0.949; HC3 95% CI, −0.983 to −0.915; *p* < 0.001; adjusted R^2^ = 0.931). Family-clustered confidence intervals were similar. Associations remained strong across all four domains (Table 2; Figure 4). In separate adjusted models, standardized coefficients for plasma melatonin and aMT6s rank percentiles were −0.877 (HC3 95% CI, −0.918 to −0.835) and −0.949 (HC3 95% CI, −0.998 to −0.899), respectively.

### 3.4. Robustness, Collinearity, and Missing-Data Sensitivity

The association was not materially changed by alternative score construction. PCA among 308 complete marker cases produced a first component explaining 75.5% of marker variance; its correlation with the melatonergic axis was −0.947. After 1st/99th percentile winsorization, the axis-integrated burden correlation was −0.944 and the HC3-adjusted coefficient was −0.950. Leave-one-domain-out correlations ranged from −0.951 to −0.915, and leave-one-marker-out correlations ranged from −0.948 to −0.943. These analyses show that no single domain or marker solely generated the primary association, although they do not externally validate the score.

Variance inflation factors for the melatonergic-axis score, age, sex, and BMI ranged from 1.04 to 1.18, providing no evidence of problematic collinearity among regression predictors. The 64 adults omitted from the BMI-adjusted model all lacked BMI; one also lacked sex. Complete cases and omitted participants did not differ significantly in age, sex distribution, plasma melatonin, aMT6s, axis score, or integrated burden, although such comparisons cannot exclude selection bias. The integrated-burden coefficient was stable in the age-and-sex model (N = 313; β = −0.949), the complete-case age-sex-BMI model (N = 250; β = −0.949), and the mean-imputed BMI plus missingness-indicator model (N = 313; β = −0.951). Full results are provided in the Supplementary Analysis workbook and Tables S1–S5.

## 4. Discussion

### 4.1. Principal Findings

In this adult secondary analysis, lower relative melatonergic status was associated with a broad adverse pattern spanning inflammation, oxidative damage, antioxidant deficit, and biological aging/injury. The association persisted when plasma melatonin and aMT6s were considered separately and after winsorization, alternative PCA weighting, leave-one-domain/marker-out analyses, robust covariance estimation, and missing-BMI sensitivity models. The central result is therefore an internally coherent cross-sectional biomarker relationship, not a cancer diagnostic or prognostic model.

### 4.2. Biological Interpretation and Alternative Explanations

The direction of the association is biologically plausible because melatonin participates in mitochondrial redox control, antioxidant enzyme regulation, and immune signaling, while chronic inflammation and oxidative stress can accompany aging and tissue injury [3,4,7,8,9,10,11,12,13,14]. However, plausibility does not establish causal direction. Lower melatonin could contribute to inflammatory or oxidative dysregulation; systemic illness or inflammation could suppress melatonin biology; both could reflect sleep, light, medication, metabolic, renal, or other shared determinants; or the pattern could arise partly from the structure and provenance of the biomarker block.

The magnitude of the correlations is unusually high for observational human biomarker research and requires critical interpretation. The exposure and outcome composites do not contain the same variables, so the association is not a direct mathematical identity. Nevertheless, the individual biomarkers and domain composites are themselves highly correlated, and shared assay batches, selection, preprocessing, data reconstruction, or restricted biological range could increase apparent coherence. The robustness analyses demonstrate internal stability but cannot substitute for audit of primary assay exports, sample identity, plate/batch structure, lower-limit handling, prior imputation, or replication in an independently collected cohort.

Equal weighting was intentionally transparent and avoided using a cancer outcome that was not present to optimize weights. Similar PCA, winsorized, and leave-one-out results reduce concern that a single arbitrary weight or outlying marker explains the finding. However, equal weights do not imply equal biological importance, and the integrated systemic burden score should remain an exploratory research summary rather than a clinical scale.

### 4.3. Oncology Relevance and Interpretation Boundary

The present dataset contained no participant-level microbiome, cancer, CTC, cfDNA, or ctDNA data. Therefore, the observed systemic association cannot be used to infer cancer initiation, occult malignancy, recurrence, metastasis, treatment response, or molecular residual disease. Microbiome research supports complex relationships among microbial communities, diet, inflammation, tumor biology, and treatment response, while emphasizing substantial confounding and methodological heterogeneity [15,16,17,18,19,20,21]. The current analysis cannot determine whether relative melatonergic status precedes dysbiosis, follows dysbiosis, or shares upstream determinants with it.

Liquid biopsy markers should likewise be positioned as tumor-specific downstream endpoints. CTCs and ctDNA can provide prognostic, genotyping, monitoring, or molecular residual disease information in defined cancer populations, but performance depends on tumor type, stage, assay, sampling schedule, and clinical setting [22,23,24,25,26,27,28,29,30,31,32]. The present systemic biomarkers cannot be interpreted as evidence of occult cancer or as substitutes for validated CTC/cfDNA/ctDNA assays.

### 4.4. Causality, Intervention, and Clinical Meaning

The cross-sectional design cannot show that lower melatonin causes systemic biomarker abnormalities or cancer. It also provides no evidence that melatonin supplementation would normalize the measured markers, prevent malignancy, or improve oncological outcomes. Interventional claims would require randomized or carefully controlled prospective studies with standardized formulation, dose, timing, adherence, circadian-phase assessment, safety monitoring, and prespecified clinical endpoints. No clinical cut-off or deficiency threshold was derived in this analysis.

### 4.5. Strengths and Limitations

Strengths include complementary melatonergic measures, a broad biomarker panel, transparent composite definitions, direct comparison of plasma melatonin with aMT6s, bootstrap uncertainty estimation, robust and family-clustered covariance analyses, alternative PCA weighting, leave-one-out analyses, and explicit missing-BMI sensitivity assessment. The revised framework also separates measured findings from unmeasured oncology hypotheses.

Limitations are substantial. The analysis is secondary, cross-sectional, exploratory, and unreplicated. The dataset lacks cancer outcomes, microbiome and liquid biopsy measurements, detailed recruitment criteria, comorbidities, medication and supplement use, smoking, sleep, shift work, light exposure, sampling clock time, urine-collection quality control, and complete assay provenance. BMI was missing in 64 adults. An untimed plasma melatonin measurement cannot characterize circadian phase or nocturnal amplitude. The exceptionally strong biomarker correlations require verification against primary laboratory and data-management records. Residual and unmeasured confounding remain likely, and no diagnostic accuracy, prospective predictive value, or clinical utility was established.

These limitations have specific consequences for the interpretation of the findings. First, because blood collection was not clock-timed and information on ambient light, sleep timing, season, and other circadian conditions was unavailable, plasma melatonin cannot be interpreted as a measure of circadian phase, nocturnal amplitude, or clinically defined melatonin deficiency. Second, incomplete assay metadata—including manufacturer, calibration, detection limits, analytical precision, batch or plate identifiers, storage duration, and freeze–thaw history—preclude a complete assessment of analytical validity and may have contributed to the unusually strong inter-marker coherence. Third, the absence of detailed comorbidity, medication, supplement, smoking, sleep, shift-work, light-exposure, renal-function, and hepatic-function data prevents comprehensive control of residual and unmeasured confounding. Finally, because no cancer diagnoses or outcomes, microbiome profiles, CTC counts, or cfDNA/ctDNA measurements were available, the present analysis cannot evaluate cancer risk, prognosis, recurrence, treatment response, metastasis, or liquid biopsy performance. Accordingly, the findings should be interpreted solely as an internally consistent, hypothesis-generating cross-sectional association; the inherent limitations of the source dataset cannot be retrospectively resolved and require confirmation through primary-data audit, independent replication, and prospective longitudinal validation.

### 4.6. Prospective Validation Pathway

Future studies should be preregistered and longitudinal, with an independent validation cohort. They should include clock-timed plasma or salivary melatonin, validated 24 h or first-morning aMT6s collection with completeness and creatinine checks, sleep and actigraphy data, light exposure, season, shift work, medication and supplement use, renal and hepatic function, smoking and metabolic covariates, and assay manufacturer/batch/precision records. Oncology cohorts should add prespecified endpoints such as incident cancer, recurrence, radiological progression, treatment response, minimal residual disease, and metastasis, together with standardized microbiome sequencing/metabolomics and CTC/cfDNA/ctDNA assays. Such a design could test temporal ordering, reproducibility, incremental predictive value, and whether the systemic phenotype has any clinically meaningful oncological application.

## 5. Conclusions

Lower relative plasma melatonin and urinary aMT6s were associated with a highly coordinated pattern of higher inflammatory, oxidative damage, antioxidant deficit, and biological aging/biological injury burden in this adult cross-sectional dataset. The association was internally robust across several alternative analyses, but its exceptional magnitude, incomplete assay metadata, and absence of external replication require caution. Because cancer, microbiome, CTC, cfDNA, and ctDNA endpoints were not measured, the study does not support cancer risk prediction, diagnosis, causal claims, or melatonin treatment recommendations. It defines a testable systemic biomarker hypothesis for rigorously designed prospective studies.

## Figures and Tables

**Table 1:** Selected adult cohort characteristics overall and by relative melatonergic tertile (mean ± SD unless otherwise indicated).

Variable	Overall	Lower (n = 105)	Intermediate (n = 104)	Higher (n = 105)	*p* Value
Age, years	48.99 ± 14.07	52.38 ± 14.01	51.67 ± 13.65	42.93 ± 12.62	<0.001
Female sex, n (%)	192 (61.3%)	65 (61.9%)	64 (61.5%)	63 (60.6%)	—
BMI, kg/m^2^	27.13 ± 4.03	27.41 ± 4.69	27.28 ± 3.56	26.68 ± 3.70	0.552
Plasma melatonin, pg/mL	12.90 ± 8.40	4.18 ± 1.99	12.06 ± 3.45	22.46 ± 5.20	<0.001
Urinary aMT6s, µg/24 h	19.10 ± 12.85	6.01 ± 2.29	18.32 ± 6.33	32.97 ± 9.17	<0.001
hsCRP, mg/L	2.73 ± 2.00	5.05 ± 1.42	2.17 ± 0.95	0.97 ± 0.56	<0.001
IL-6, pg/mL	2.77 ± 1.93	4.93 ± 1.61	2.19 ± 0.82	1.18 ± 0.55	<0.001
MDA, µmol/L	3.71 ± 1.18	5.00 ± 0.56	3.59 ± 0.65	2.54 ± 0.63	<0.001
8-OHdG, ng/mL	7.25 ± 2.84	10.25 ± 1.47	7.02 ± 1.68	4.49 ± 1.56	<0.001
OSI, TOS/TAC	17.27 ± 10.54	29.34 ± 6.58	14.83 ± 5.48	7.62 ± 3.75	<0.001
GSH/GSSG ratio	32.46 ± 29.21	11.32 ± 2.48	25.23 ± 12.65	60.74 ± 33.06	<0.001
NT-proBNP, pg/mL	344.74 ± 200.86	592.15 ± 58.91	300.25 ± 92.22	135.36 ± 40.41	<0.001
DunedinPACE, units/year	1.07 ± 0.19	1.31 ± 0.07	1.03 ± 0.08	0.87 ± 0.05	<0.001

aMT6s, 6-sulfatoxymelatonin; BMI, body mass index; hsCRP, high-sensitivity C-reactive protein; MDA, malondialdehyde; 8-OHdG, 8-hydroxy-2′-deoxyguanosine; OSI, oxidative stress index; TOS, total oxidant status; GSH, reduced glutathione; GSSG, oxidized glutathione. The higher-tertile female percentage uses 104 participants because one sex value was missing. NT-proBNP and DunedinPACE had n = 308 overall (104, 103, and 101 by tertile). Tertiles are within-cohort categories, not clinical reference groups.

**Table 2:** Adjusted association of the relative melatonergic-axis score with systemic biomarker burden.

Outcome	N	β Per 1-SD Higher Axis	HC3 95% CI	Family-Clustered 95% CI	*p* Value	Adjusted R^2^
Inflammation	250	−0.889	−0.935 to −0.842	−0.935 to −0.842	<0.001	0.856
Oxidative damage	250	−0.957	−0.997 to −0.917	−0.996 to −0.919	<0.001	0.895
Antioxidant deficit	250	−0.933	−0.986 to −0.880	−0.982 to −0.883	<0.001	0.842
Biological aging/injury	250	−0.909	−0.935 to −0.882	−0.934 to −0.883	<0.001	0.951
Integrated systemic burden	250	−0.949	−0.983 to −0.915	−0.982 to −0.916	<0.001	0.931

Coefficients represent standardized change in each outcome per 1 SD higher melatonergic-axis score. Models were adjusted for age, sex, and BMI. HC3 confidence intervals are heteroskedasticity-robust; family-clustered intervals use the source family identifier.

## Data Availability

Aggregate results and analysis summaries are provided in the Supplementary Materials. Deidentified source data may be available from the corresponding author upon reasonable request and subject to privacy, consent, institutional, and ethical restrictions.

## Supplementary Materials

The following supporting information can be downloaded at: https://www.mdpi.com/article/10.3390/cancers18162597/s1. The file cancers-18-02597-supplementary.xlsx includes cohort flow, tertile summaries, composite correlations, conventional and robust adjusted models, alternative composite constructions, missing-data sensitivity analyses, variable definitions, methodological limitations, analysis provenance, and the numeric derivations used for Figure 2, Figure 3 and Figure 4. No direct identifiers or individual-level data are included.
